# Functional knockout of long non-coding RNAs with genome editing

**DOI:** 10.3389/fgene.2023.1242129

**Published:** 2023-08-29

**Authors:** Qing Rex Lyu, Shikuan Zhang, Zhe Zhang, Zhiyu Tang

**Affiliations:** ^1^ Medical Research Center, Chongqing General Hospital, Chongqing, China; ^2^ Chongqing Academy of Medical Sciences, Chongqing, China; ^3^ Key Lab in Healthy Science and Technology of Shenzhen, Tsinghua Shenzhen International Graduate School, Shenzhen, China; ^4^ Department of Chinese Medical Gastrointestinal of China-Japan Friendship Hospital, Beijing, China

**Keywords:** long non-coding RNA, CRISPR-Cas9, functional knockout, genome editing, methodology

## Abstract

An effective loss-of-function study is necessary to investigate the biological function of long non-coding RNA (lncRNA). Various approaches are available, including RNA silencing, antisense oligos, and CRISPR-based genome editing. CRISPR-based genome editing is the most widely used for inactivating lncRNA function at the genomic level. Knocking out the lncRNA function can be achieved by removing the promoter and the first exon (PE1), introducing pre-termination poly(A) signals, or deleting the entire locus, unlike frameshift strategies used for messenger RNA (mRNA). However, the intricate genomic interplay between lncRNA and neighbor genes makes it challenging to interpret lncRNA function accurately. This article discusses the advantages and disadvantages of each lncRNA knockout method and envisions the potential future directions to facilitate lncRNA functional study.

## Introduction

Long non-coding RNA (lncRNA) is a type of RNA transcript that is over 200 nucleotides long and lacks protein-coding potential. Like messenger RNA (mRNA), lncRNA is transcribed mainly by RNA polymerase II and undergoes post-transcriptional modifications (Nojima and Proudfoot, 2022). The use of high-throughput RNA transcript sequencing has allowed for identifying numerous lncRNAs. Despite being regarded as “junk” transcripts for a long time, the functions of lncRNAs are still not fully understood. However, many functional lncRNAs have been characterized in recent decades, demonstrating novel gene regulatory patterns. They have ushered in a new paradigm in the field (Rinn and Chang, 2012). Loss-of-function studies are a critical method for investigating the function of lncRNAs *in vitro* and *in vivo*. While deleting the promoter and first exon of lncRNA has been extensively utilized and demonstrated efficacy in deactivating lncRNA function, the intricate nature of lncRNA gene loci and their interactions with nearby or overlapping genes pose challenges in achieving precise lncRNA knockouts without affecting the expression of nearby or overlapping genes (Li Z. et al., 2021). In this article, we first introduce the biogenesis and central functional patterns of lncRNAs. We then summarize the prevailing techniques and strategies for knocking out or inactivating lncRNA function. Finally, we discuss the future directions for lncRNA functional inactivation and provide insights into practical approaches aimed at expediting lncRNA functional studies, from individual studies to high-throughput screening.

## The biogenesis and function of lncRNA

Most lncRNAs are transcribed by RNA polymerase II, which undergoes post-transcriptional modifications such as 5′capping, 3′poly(A) tailing, and intron splicing (Rinn and Chang, 2012). LncRNAs can perform biological functions via various mechanisms, either by being exported to the cytoplasm or retained within the nucleus (Rinn and Chang, 2012). LncRNAs can be categorized into two classes based on their role: sequence-dependent and sequence-independent. The sequence-dependent role of lncRNAs, known as ceRNA (competing endogenous RNA), has been well-studied. LncRNAs can bind with microRNAs via Watson-Crick base-pairing, acting as a “miRNA sponge” to neutralize targeting miRNA carrying a specific “seed region.” (Conte et al., 2021; Zhang et al., 2022). As a result, the mRNA targeted by the endogenous miRNA is “released” from miRNA repression, leading to the recovery of translational levels (Tay et al., 2014). The contribution of ceRNA to gene regulation has been controversial due to the promiscuous miRNA-lncRNA-mRNA interplay, which makes quantitative analysis difficult (Tay et al., 2014). Another important sequence-dependent function of lncRNAs is serving as a “guide.” In this mechanism, a partial sequence on the lncRNA can bind to genomic DNA via sequence-specific pairing.

In contrast, another part of the lncRNA can form a specific tertiary structure and associate with a protein via electrostatic force. A well-known example of this category is the small guide RNA (sgRNA) and Cas proteins (Jinek et al., 2012; Cong et al., 2013), with derivatives such as prime editing guide RNA (pegRNA) and gene-modified prime editors (Anzalone et al., 2019; Anzalone et al., 2020). Another sequence-dependent function of lncRNAs is encoding small or micro peptides. For instance, Anderson et al. discovered that the *LINC00948* lncRNA encodes a peptide called MLN, which interacts with SERCA to regulate the phenotype in skeletal muscle (Anderson et al., 2015).

Most characterized lncRNAs function sequence independently by forming tertiary structures and interacting with proteins as a scaffold (Yoon et al., 2013; Andric and Rougemaille, 2021). Dozens of protein partners of lncRNAs have been reported (Gerstberger et al., 2014), among which the PRC2 (Polycomb Repressive Complex 2) complex has been well-studied. The PRC2 core complex consists of EED, SUZ12, EZH1, EZH2, etc. (Blackledge and Klose, 2021). LncRNAs such as *XIST*, *HOTAIR*, *ANRIL*, etc., interact with PRC2 components in the nucleus, regulating H3K27 methylation and gene silencing. These lncRNAs play an essential role in dosage compensation, imprinting, and histone accessibility (Yoon et al., 2013). LncRNAs can also associate with proteins in the cytosol. For example, the vascular smooth muscle cell and endothelial cell-enriched lncRNA, *SENCR*, interacts with cytoskeletal-associated protein (CKAP4) and modulates the surface localization of cadherin 5 (CDH5) (Lyu et al., 2019). This interaction plays a critical role in maintaining homeostasis and integrity of the vascular endothelial monolayer (Lyu et al., 2019). Another noteworthy lncRNA functioning pattern is *in-cis* interacting lncRNA. The lncRNA *Upperhand* (*Uph*) and the protein-coding gene Hand2 localize on chromosome 8 in the mouse genome in a divergent direction (Anderson et al., 2016). There is merely a 150 bp gap between the transcription start site (TSS) between *Uph* and *Hand2*. *Uph* transcription processing establishes a permissive chromatin status and therefore benefits *Hand2* transcription initiation. The blockade of *Uph* transcription, but not the knockdown of *Uph* transcripts, compromises *Hand2* expression, suggesting that *Uph* plays an important role in *Hand2* transcription regulation (Anderson et al., 2016).

## The genomic complexity of lncRNA

Most lncRNAs are multi-exons and undergo maturation processes like mRNAs: transcribed by the RNA polymerase II, primary lncRNA transcripts are 5′capped, 3′polyadenylated, and introns are removed (St Laurent et al., 2015). Different from mRNAs exported to the cytoplasm for protein synthesis, a portion of lncRNAs retained in the nucleus and play essential roles in gene regulation (St Laurent et al., 2015). For example, the inactive X chromosome-specific transcript, *XIST*, was discovered in the cell nucleus that dominates the X-inactivation by silencing one of the two female X chromosomes and preventing the dosage effect (Chao et al., 2002; Lee, 2012). Here arises a puzzle about how lncRNAs retained in the cell nucleus, unlike their protein-coding counterparts. Yin et al. reported that U1 snRNP regulates the chromatin retention of non-coding RNAs and revealed that mutation of the 3′splicing site causes nuclear localization and performs *cis*-regulation on mRNA transcription (Yin et al., 2020). However, the regulatory function of nuclear lncRNAs is far from clear, partially attributed to the lack of understanding of RNA-binding proteins (RBPs) since RBPs mediate most lncRNA functions. It is estimated that there are ∼1,500 RBPs encoded by the human genome (Gerstberger et al., 2014), whereas the actual number could be significantly more. Lately, the genome-scale investigation of RNA-binding proteins unveiled the regulatory pattern of lncRNA-protein, suggesting a novel paradigm for lncRNA study (Xiao et al., 2019; Van Nostrand et al., 2020).

**FIGURE 1:**
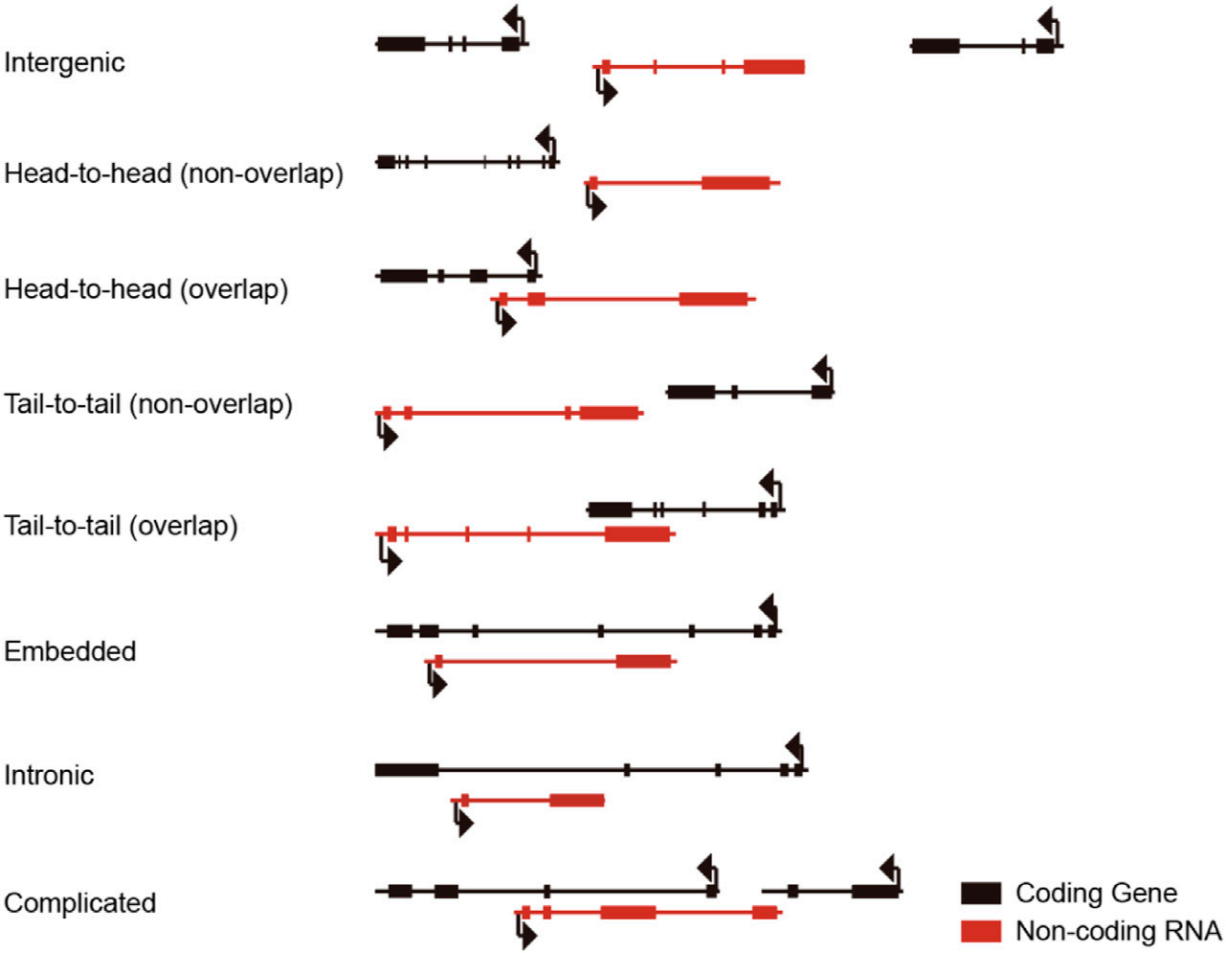
Relative localization of lncRNA and adjacent protein-coding genes.

Bearing multiple introns makes lncRNA occupy a large piece of genomic DNA space for accommodating full-length lncRNA. The longer the lncRNA primary transcript is, the more likely it overlaps and intervenes with another mRNA, lncRNA, or snoRNA coding sequence. Thus, lncRNAs were categorized into long intergenic non-coding RNA, head-to-head overlap, head-to-head non-overlap, tail-to-tail overlap, tail-to-tail non-overlap, embedded, intronic, and complicated form lncRNA according to the relative genomic localization of lncRNA to the nearby protein-coding gene (Figure 1). Due to the complexity of the genomic locus of lncRNAs, it is a big challenge to inactive a target lncRNA without perturbing adjacent or overlapped transcript counterparts, hampering the functional study of lncRNAs.

## The strategies for lncRNA functional knockout

Multiple strategies have been established to inactivate the lncRNA function via inheritable and uninheritable approaches, including but not limited to siRNA duplex silencing, antisense oligonucleotides (ASO) inhibition, and CRISPR-based genome editing (Miano et al., 2019). CRISPR-based genome editing is the primary technique resolution to create stable and transferrable lncRNA knockout cell lines or animals. Here, we summarized the overall knockout or knockdown strategies and discussed the advantages and disadvantages of each method.

### 1 Removing the promoter and the first exon of lncRNA

**FIGURE 2:**
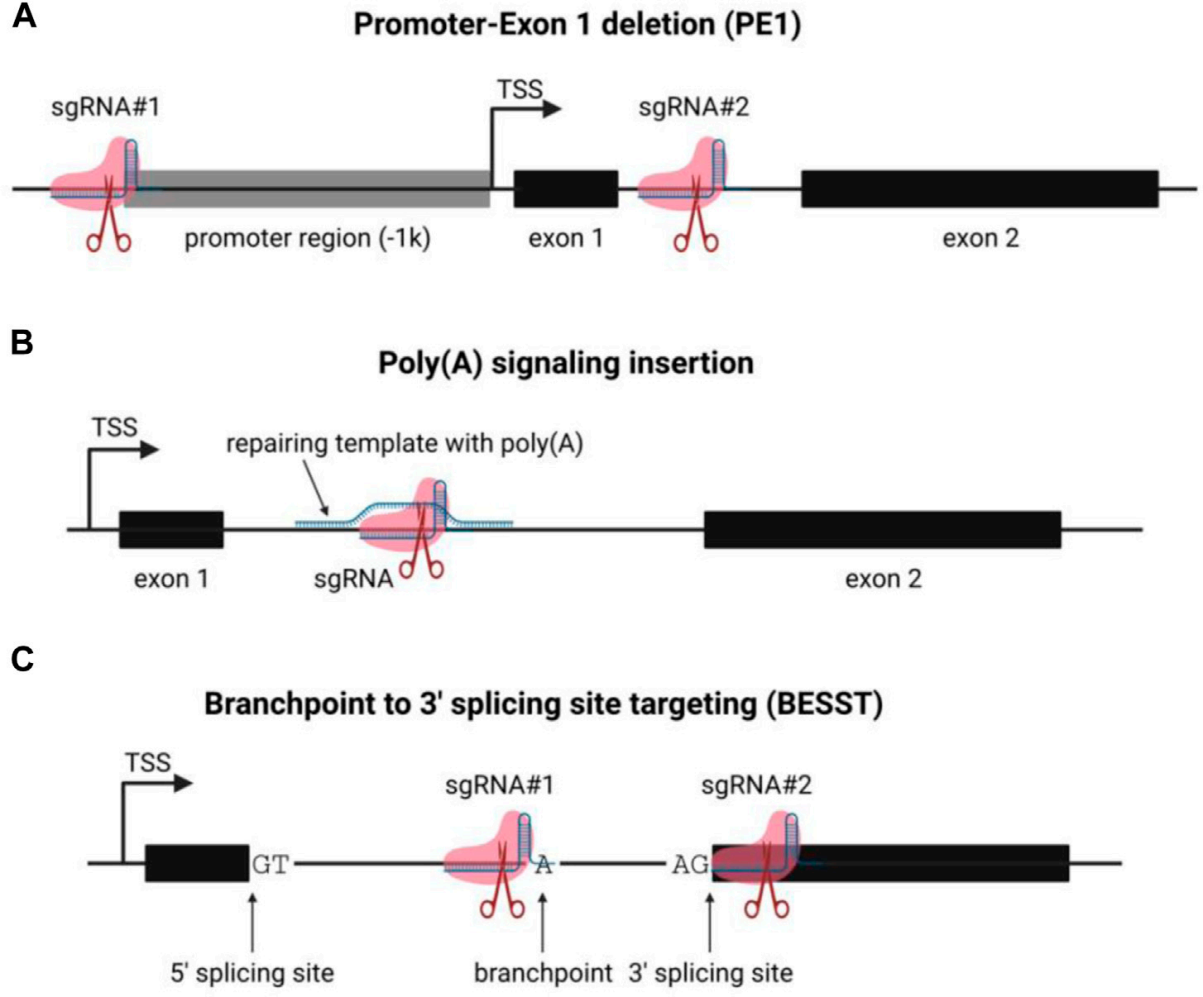
Schematic of lncRNA functional knockout strategies. **(A)** the schematic of promoter-exon1 deletion (PE) lncRNA knockout strategy; **(B)** the schematic of poly(A) signal insertion for lncRNA knockout; **(C)** the schematic of BESST lncRNA knockout method.

Like mRNA, most lncRNAs were transcribed by RNA polymerase II and regulated by the accessibility of the chromatin state and the transcriptional factors that directly bind to the DNA duplex to recruit the initiation complex (St Laurent et al., 2015). Thus, a widely used strategy to inactivate lncRNA expression is to remove the promoter region from the transcription start site (TSS) to 1,000–2000 base pair upstream (Figure 2A) (Zhang et al., 2012; Ho et al., 2015; Zhen et al., 2017). Provided that most lncRNAs are multi-exon, the first exon of the target lncRNA is usually excised to ensure the inhibitory effect (Aparicio-Prat et al., 2015; Li et al., 2021). In brief, two sgRNAs are designed using sequencing flanking (±100 bp) target areas (-1k upstream of TSS, the 5′splicing site of the first intron). The candidate sequences are analyzed and optimized by the on-targeting and off-targeting computational algorithms. The sgRNAs are synthesized as mature crRNA (Jinek et al., 2012) or inserted into the sgRNA expression cassette plasmids (Ran et al., 2013). The delivery of Cas9 protein can be achieved via various approaches, viral-based vectors, *in vitro* transcribed (IVT) RNA, and purified protein (Luther et al., 2018).

The genome editing method has been comprehensively described (Rosenlund et al., 2021), and we are not going through all the details here. The advantages of this strategy include 1) simple: only two sgRNAs and Cas9 to be delivered, the genome editing efficiency is good; 2) excellent inhibition: since the driver of the target transcript is wholly removed, the target lncRNA is very unlikely to be expressed; 3) friendly to genotyping: the editing generates an approximately a thousand base pair gap in the genomic DNA, which is convenient to be detected via genotyping PCR and agarose gel electrophoresis. However, the flaw of this method is also significant, the apparent genome disturbance. Many lncRNA loci are close, overlap, or included in the genomic loci of another transcript, typically protein-coding genes (Sigova et al., 2013). Thus, removing the promoter and the first exon of the lncRNA is likely to delete the protein-coding sequence of transcripts. It causes direct compromise of the protein-coding genes and transcriptome interference and phenotype change. The collateral intervention could mislead the researcher and mask the natural biological function of the target lncRNA.

### 2 The insertion of pre-termination poly(A) signal

Another widely-used method to inactivate the lncRNA function is to introduce a poly(A) signal downstream of the TSS, which causes the detachment of RNA polymerase II from the DNA strand and disassembly (Figure 2B) (Liu et al., 2017). Only one sgRNA is required for this approach to generate a double-strand break (DSB) by Cas9 protein. Notably, a repair template (single-strand DNA is preferred) is needed to serve as an HDR (homology-directed repair) template to introduce the poly(A) signal containing codons via homologous recombination (Powell et al., 2021). Notably, this strategy minimizes the perturbance of the genome structure: only a few hundred nucleotides are inserted (Liu et al., 2017; Lavalou et al., 2019; Powell et al., 2021). However, a few significant drawbacks are preventing it from the extensive application: 1) Low efficiency: insertion of poly(A) signal requires 3-component (sgRNA, Cas9, HDR template) CRISPR-based genome editing, compared to the 2-component (sgRNA, Cas9) strategy, the overall genome editing efficiency is limited by the recombination efficiency of the HDR template. 2) Alternative TSS: Like mRNAs, lncRNAs undergo alternative splicing and generate multiple splicing variants. If a lncRNA transcript has an alternative TSS, the knockout could fail if the poly(A) signal insertion site is upstream of the second alternative TSS. A truncated lncRNA transcript will be transcribed if the poly(A) signal insertion is too far from the TSS (McDonel and Guttman, 2019). Despite the low efficiency, poly(A) signal insertion is still an applicable alternative for terminating RNA transcript *in vitro* and *in vivo*.

### 3 The branchpoint to 3′splicing site targeting (BESST) knockout strategy

It is well-established that lncRNAs reside in intricate gene contexts within the mammalian genome. Numerous lncRNAs are in close proximity to neighboring protein-coding genes, which can be categorized into various natural antisense transcripts (NATs), including head-to-head, tail-to-tail, and entirely overlapped (Reis and Poirier, 2021). Therefore, it is highly probable that the conventional CRISPR-based gene knockout strategy, which involves removing the promoter and first exon of an RNA transcript, will affect the neighboring protein-coding gene on the same or opposite strand. Goyal et al. reported that nearly 60 percent of 15,929 lncRNAs were not in secure situations for CRISPR-based gene editing (Goyal et al., 2017), indicating that deletion of lncRNA almost certainly results in the inactivation of protein-coding genes. The BESST (branchpoint to 3′splicing site targeting) gene knockout strategy is a recently described method for inactivating lncRNA and protein-coding genes by removing the branchpoint to the 3′splicing site of the last intron within a compact 18–44 nt region (Figure 2C) (Zhang et al., 2023). The BESST removes the DNA segment from the branchpoint to the 3′splicing site in the last exon of the target lncRNA, resulting in the intron retention and nuclear retention of the target RNA. Further research revealed that the BESST gene knockout induces lncRNA nuclear degradation by decapping via NCBP1-dependent mechanism and poly(A) deadenylation via PABPN1-mediated and CNOT7/CNOT8-dependent mechanism. Notably, the BESST knockout was validated to also function in multi-exon protein-coding genes, providing an alternative method for studying gene function. Another important aspect of the BESST knockout method is that it could facilitate researchers to determine the underlying mechanism of lncRNA. LncRNA has multiple modes of functioning, including *trans-* and *cis*-action. The BESST knockout technique could distinguish between sequence-dependence and transcription-dependence in the mechanism of lncRNAs across their genomic loci (McDonel and Guttman, 2019; Zhang et al., 2023). However, since there is no intron to deal with, we were unable to disregard the BESST application’s limitations in single-exon RNA transcripts. In brief, the emergence of the BESST knockout strategy provides a convenient tool to inactivate multi-exon RNA transcripts with minimal genomic DNA perturbation, which could be applied to precisely identify gene function via high throughput screening. Notably, the efficiency and feasibility of BESST will be further enhanced by using optimized Cas proteins which allow less stringent PAM (protospacer-adjacent motif) sequence (Walton et al., 2020).

### 4 The uninheritable approach

Besides the CRISPR-based lncRNA knockout strategy, other methods cannot be inherited *in vivo*, including RNA silencing and antisense oligo (Stojic et al., 2018). RNA silencing is the knockdown gene approach using synthesized siRNA duplex or viral-based shRNA expression vector (Elbashir et al., 2001; Rao et al., 2009). RNA silencing is effective for knocking down protein-encoding genes, whereas it only works for a portion of lncRNA. The molecular principle of RNA silencing is the siRNA/shRNA duplex being loaded into the RISC complex and functions as a guide to associate with target mRNA with Watson-Crick pairing and initiating RNA degradation (Rao et al., 2009). However, RNA silencing does not work for nuclear lncRNA since there is no RISC complex distribution in the cell nucleus (Zeng and Cullen, 2002; Lennox and Behlke, 2016). Antisense oligonucleotide (ASO) is a short single-strand DNA that targets lncRNA in a sequence-specific manner and triggers RNase H activity for degradation (Bennett, 2019). However, ASO can only be delivered by transfection and not be loaded into a plasmid, establishing a stable expression line as shRNA. So, the animal application is limited even if the ASO showed superior efficiency in nuclear lncRNA knockdown. Cas13 is a newly emerged genetic tool that directly edits RNA transcript efficiently (Pickar-Oliver and Gersbach, 2019), whereas the uninheritable attribute of the method dramatically limits its application in animal study. The pros and cons of the aforementioned methodologies are summarized in Table 1.

**TABLE 1:** The merit and demerit of the gene knockout/knockdown methods.

Method	Mechanism	Merit	Demerit	FDA approved drugs
PE1	Remove promoter and suppress binding of transcriptional machinery	1) Straightforward for sgRNA designing spanning promoter and first exon	1) Disturb adjacent genes and cause unintended gene suppression, not suitable for clinical objectives	N/A
		2) Work for both nuclear and cytosolic lncRNAs	2) remove a large piece of DNA	
		3) Can be transferred to descendants		
BESST	Cause nuclear retention of target lncRNA, induce intranuclear RNA degradation	1) Straightforward for designing targeted two sgRNAs	1) Not work for single exon RNA transcripts	N/A
		2) High efficiency	2) PAM-less Cas protein preferred	
		3) work for nuclear and cytosolic lncRNAs		
		4) inheritable		
Poly(A) Insertion	Pre-terminate RNA transcription by Poly(A) signals	1) Straightforward for designing targeted one sgRNA	1) 3-component CRISPR editing	N/A
		2) work for nuclear and cytosolic lncRNAs	2) Low efficiency	
siRNA	Cleave and degrade target RNA with a sequence-dependent manner	1) Straightforward for designing siRNA duplexes	1) Transient transfection	1) Patisiran, hereditary transthyretin amyloidosis (hTTA)
		2) Mature to deliver by various transfection strategies	2) Off-targeting	2) Givosiran, acute hepatic porphyrias
		3) Work only for cytosolic lncRNAs		3) Umasiran, primary hyperoxaluria type 1 (PH1)
		4) Apply clinically		4) Inelisiran, hypercholesterolemia
Lentiviral shRNA	Same with siRNA	1) Straightforward for designing siRNA/shRNA	1) The inhibition rate might reduce over time	N/A
		2) Easy to deliver via lentiviral inoculation	2) Off-targeting	
		3) Work only for cytosolic lncRNAs		
ASO	Intranuclear cleavage by RNase H with a sequence-dependent manner	1) Straightforward for designing	1) Transient transfection	1) Formivirsen, CMV
		2) works only for nuclear lncRNAs	2) Trigger intracellular immune response	2) Mipomersen, hypercholesterolemia
		3) Apply clinically		3) Eleplinsen, Duchenne muscular dystrophy (DMD)
				4) Musinersen, spinal muscular atrophy (SMA)
				5) Nolersen, hTTA
				6) Golodirsen, DMD
				7) Viltolarsen, DMD
				8) Casimersen, DMD

## Perspectives

LncRNAs are essential regulatory molecules in cells, but the majority of lncRNAs’ biological functions are unknown. This is largely due to the fact that current functional studies are restricted to analyzing individual lncRNAs, which is laborious and inefficient. In recent years, the CRISPR-based genome-scale knockout screening and transcriptional activation method for protein-coding genes were established (Fox et al., 2018). Subsequently, the CRISPR-based genome-scale deletion screening of human lncRNAs was also conducted (Zhu et al., 2016). Due to technical limitations, these early initiatives can only investigate intergenic lncRNAs and not natural antisense lncRNAs, since large-piece DNA deletion result in unintended protein-coding or non-coding RNA expression intervention and lead to unpredictable effects. The emergence of precise and genome-wide lncRNA functional knockout methodology would no doubt expedite the understanding of lncRNA more efficiently. It will assist researchers to comprehend the importance of non-coding RNAs in various biological and pathological processes and could also be utilized for developing potential therapeutical interventions.

Different from proteins, the biological function of lncRNAs is mainly exerted via interacting with proteins (Kopp and Mendell, 2018). Rinn et al. introduced diverse patterns of lncRNAs functioning with their protein partners, including decoy, scaffold, guide, and enhancer (Rinn and Chang, 2012). Nevertheless, the association between lncRNA and protein is promiscuous since the RNA-protein interaction is mediated by the non-covalent bond and depended on the RNA tertiary structure, which is flexible (Gruber et al., 2008; Ferre et al., 2016). The tertiary structure of RNA is more dynamic than that of proteins and is influenced by the transcriptome, interacting proteins, ion strength, and so on (Jaeger et al., 1989; Li and Aviran, 2018). It is difficult and pointless to determine the “true” RNA tertiary structure of a target lncRNA in the complex biophysical environment in living cells because different cell types exhibit distinct transcriptomes and proteomes (Li and Aviran, 2018; Sanchez de Groot et al., 2019; Wang et al., 2021). However, we can elucidate the biological function of RNA via the aspect of the interacting proteins, which can be accomplished by a variety of technical resolutions (McHugh et al., 2014; Ramanathan et al., 2019). For example, the PAR-CLIP (photoactivatable ribonucleoside enhanced crosslinked immunoprecipitation) is able to pinpoint the exact nucleotides on RNA transcripts that are directly interacting with binding proteins by *in vivo* labeling nascent RNAs with photoreactive nucleosides 4-thiouridine (4-SU) or 6-thioguanosine (6SG) (Hafner et al., 2010). The information on direct protein-binding nucleotides revealed by PAR-CLIP could also be utilized for calculating the potential RNA tertiary structure in the future.

The lncRNA expression is sophisticatedly coordinated in a spatiotemporal manner. There are two fundamental components of spatiotemporal regulation: where and when. LncRNAs are expressed in different cells, especially during development. For instance, Kim and colleagues reported that lncRNA *Moshe* is detected in the cardiac mesoderm stage (E8.5 to E9.5) along with the expression of *Gata6*, and continues to be expressed and increases in the atrioventricular septum (E12.5) (Kim et al., 2021). Knock-down of *Moshe* causes transcriptional suppression of *Nkx2.5* and increases the expression of second heart field (SHF) lineage genes, which may play a critical role in ASD. Another level of spatial expression of lncRNAs is subcellular localization. LncRNAs exhibit distinguished biological functional and underlying mechanisms in different cell compartments. Localized lncRNAs in the cell nucleus are primarily involved in epigenetic and transcriptional regulation, whereas cytoplasmic lncRNAs are typically involved in post-transcriptional regulation, protein-protein interactions, etc. (Yao et al., 2019; Gao et al., 2020). Accordingly, a new technique, APEX-seq, was developed which can dissect the function of lncRNA in different subcellar localizations, enabling a comprehensive understanding of RNA spatiotemporal regulation (Fazal et al., 2019).

Last, but not least, the expanding range of RNA-binding proteins (RBPs) also broadens our view of lncRNA functioning. RBPs are proteins containing one or more RNA binding domains (RBDs) that can directly interact with RNA strands and alter their structure and function (Hentze et al., 2018). Several hundred RBPs have been identified over the years, but studies have found RBPs without conventional RBDs, suggesting the number of RBPs is underestimated (Hentze and Argos, 1991; Hentze, 1994). Notably, the unconventional RBPs introduce the subject of phase condensation or phase separation, another crucial phenomenon in cells. A growing body of research has shown that lncRNAs and unconventional RBPs, such as paraspeckles in the nucleus and processing bodies in the cytoplasm, are crucial for organizing nonmembrane organelles and controlling gene expression (Fox et al., 2018; Elguindy and Mendell, 2021; Hirose et al., 2023). The role of lncRNA in the formation of non-membrane organelles and how it supports specific biological processes remain largely unknown. Thus, a comprehensive multi-omics strategy is required to reveal the spatiotemporal function of lncRNA in various biological and pathological processes.
